# InClust+: the deep generative framework with mask modules for multimodal data integration, imputation, and cross-modal generation

**DOI:** 10.1186/s12859-024-05656-2

**Published:** 2024-01-24

**Authors:** Lifei Wang, Rui Nie, Xuexia Miao, Yankai Cai, Anqi Wang, Hanwen Zhang, Jiang Zhang, Jun Cai

**Affiliations:** 1 Shulan (Hangzhou) Hospital, Affiliated to Zhejiang Shuren University Shulan International Medical College, Hangzhou, China; 2 China National Center for Bioinformation, Beijing, China; 3 Key Laboratory of Genomic and Precision Medicine, Beijing Institute of Genomics, Chinese Academy of Sciences, Beijing, 100101 China; 4 University of Chinese Academy of Sciences, Beijing, 100049 China; 5 School of Economic and Management, China University of Geoscience, Wuhan, China; 6 School of Systems Science, Beijing Normal University, Beijing, 100875 China

**Keywords:** Deep generative framework with mask modules, Multi-omics, Data integration, Cross-modal imputation, Cross-modal generation

## Abstract

**Background:**

With the development of single-cell technology, many cell traits can be measured. Furthermore, the multi-omics profiling technology could jointly measure two or more traits in a single cell simultaneously. In order to process the various data accumulated rapidly, computational methods for multimodal data integration are needed.

**Results:**

Here, we present inClust+, a deep generative framework for the multi-omics. It’s built on previous inClust that is specific for transcriptome data, and augmented with two mask modules designed for multimodal data processing: an input-mask module in front of the encoder and an output-mask module behind the decoder. InClust+ was first used to integrate scRNA-seq and MERFISH data from similar cell populations, and to impute MERFISH data based on scRNA-seq data. Then, inClust+ was shown to have the capability to integrate the multimodal data (e.g. tri-modal data with gene expression, chromatin accessibility and protein abundance) with batch effect. Finally, inClust+ was used to integrate an unlabeled monomodal scRNA-seq dataset and two labeled multimodal CITE-seq datasets, transfer labels from CITE-seq datasets to scRNA-seq dataset, and generate the missing modality of protein abundance in monomodal scRNA-seq data. In the above examples, the performance of inClust+ is better than or comparable to the most recent tools in the corresponding task.

**Conclusions:**

The inClust+ is a suitable framework for handling multimodal data. Meanwhile, the successful implementation of mask in inClust+ means that it can be applied to other deep learning methods with similar encoder-decoder architecture to broaden the application scope of these models.

**Supplementary Information:**

The online version contains supplementary material available at 10.1186/s12859-024-05656-2.

## Introduction

Recently, the progress of single-cell technology (e.g. single-cell RNA sequencing (scRNA-seq) [1, 2], single-cell assay for transposase-accessible chromatin sequencing (scATAC-seq) [3] and single-cell bisulfite sequencing (scBS-seq) [4]) makes it possible to obtain a variety of traits in a single cell. These single-cell methods have greatly promoted our understanding of cells. As a result, the heterogeneity of cell population was revealed [2, 5], the trajectory of cell development was inferred [6], and the gene regulatory network was reconstructed [7]. But data collected in one modality just represents a limited side view of the cell state. In order to obtain more holistic and comprehensive information, data from different modalities need to be integrated together to better reveal the biological significance of the data.

Initially, the integration of data from different modalities was accomplished by computational approaches [8, 9]. Then, multi-omics profiling technology that could jointly profile multiple traits in a single cell was developed [10]. Several methods (e.g. SNARE-seq [11], sci-CAR [12], Paired-seq [13], and SHAER-seq [14]) could simultaneously measure the gene expression and chromatin accessibility in a single cell. Cellular indexing of Transcriptomes and Epitopes by sequencing (CITE-seq) could jointly profile the gene expression and a panel of cell surface proteins [15, 16]. The scNMT-seq could profile chromatin accessibility, DNA methylation, and transcription in single cells at one time [17].

Several computational approaches have been developed to process and integrate data in single-cell analysis. Some are universal methods that could handle situations with either multiple monomodal data profiled from different cells in similar populations or multimodal data extracted from single cells [18]. Others are specially designed for processing data generated by multi-omics profiling technology [19, 20]. Usually, for data generated by multi-omics profiling technology, the information from different modalities is coded by different encoders first, and then integrated in the latent space. The one-to-one correspondence between encoders and modalities is mainly due to the fact that data from different modalities have different data formats and lengths, which can’t be encoded by the same encoder. Following the encoding, the coded information from different modalities is integrated in the latent space, through adversarial loss [21], mixture of expert [22], attention-transfer [23], regulatory interaction information [24], and so on. After integrating data from different modalities, data from one modality could be used to impute data from another modality [18]. Meanwhile, integration of multimodal data makes the translation between different modalities possible [21]. Furthermore, the multimodal data could be used as a reference to generate data of the missing modality in monomodal data [25].

**Fig. 1:**
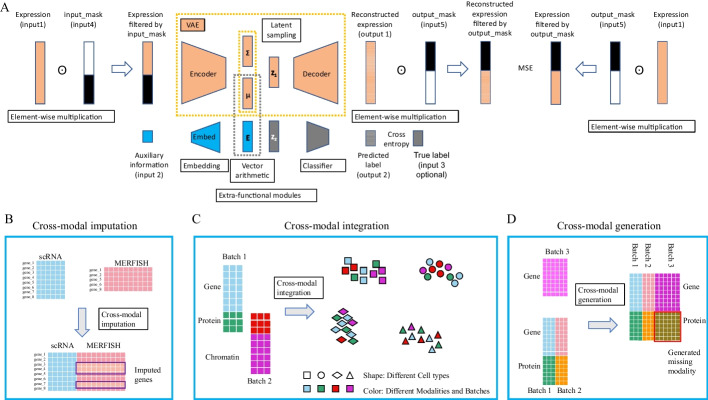
Architecture of inClust+ and its application. **A** The architecture of inClust+. InClust+ is based on inClust, with a VAE backbone (an encoder, a sampling part, and a decoder) and three built-in functional modules (an embedding layer embedded auxiliary information into the latent space, the vector arithmetic part performs information integration, a classifier cluster cells into groups). In addition, two mask modules designed for multimodal data processing are augmented to original inClust: an input-mask module in front of the encoder and an output-mask module behind the decoder. Each mask module is used to filter out unwanted values, and achieve multimodal integration and translation. **B**–**D** The application of inClust+. **B** Cross modal imputation by inClust+. There are two datasets, one from scRNA-seq (blue) and the other from MERFISH with some missing genes (red). InClust+ could impute the missing genes in MERFISH dataset (in the purple box) by referring to the scRNA-seq dataset. **C** Cross modal integration by inClust+. There are two paired datasets, one contains gene expression (blue) and protein abundance (green), and the other contains protein abundance (red) and chromatin accessibility (purple). InClust+ could integrate all three modalities from the two datasets. **D** Cross modal generation by inClust+. There are three datasets, two of which are paired datasets with gene expression (blue and red) and protein abundance (green and orange). The third one is a monomodal dataset with only gene expression data (purple). InClust+ could generate the protein abundance data (in the red box) for the third monomodal dataset by referring to the paired datasets

Previously, we presented inClust (integrated clustering), a flexible all-in deep generative framework for transcriptome data [26]. Here, we extended the inClust by adding two new modules, namely, the input-mask module in front of encoder and the output-mask module behind decoder (Fig. 1A). We named the augmented inClust as inClust+, and demonstrated that it could complete not only data integration but also gene imputation by the merits of mask modules (Fig. 1B). Furthermore, for multimodal datasets with different data types for each modality, inClust+ adopted the architecture of stacked encoder and decoder, which was used in conjunction with the mask modules. Therefore, inClust+ could integrate multimodal data, where different modalities have different data types (Fig. 1C). Finally, inClust+ with stacked encoder-decoder and mask modules was used to tackle the problem of cross modal generation (Fig. 1D). All results show that inClust+ is an ideal tool to deal with multimodal data, and adding masks is a suitable way to model augmentation in the field of multi-omics.

## Results

### InClust+ imputes genes for MERFISH data after integrating scRNA-seq and MERFISH data

**Fig. 2:**
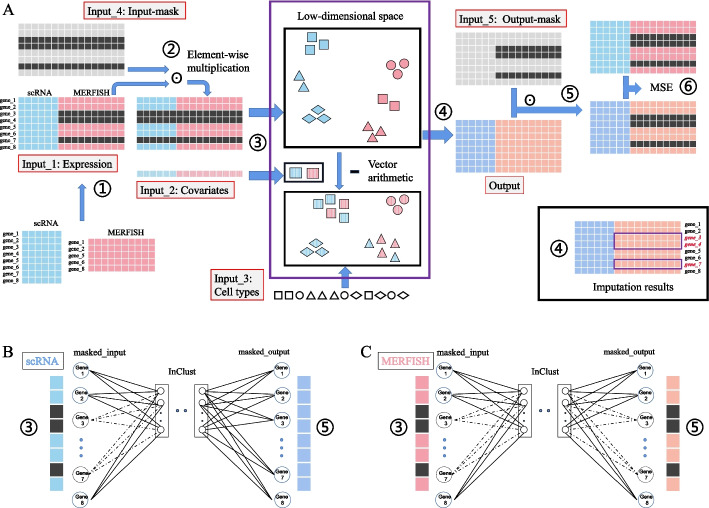
The diagram for integration of multiple monomodal (unpaired) data and subsequently gene imputation by inClust+ (see details in Additional file 2). **A** The workflow. Training: ①Generation of the training dataset. The data from scRNA-seq and MERFISH were aligned with common genes, and the missing scRNA-seq-specific genes in MERFISH data were filled with 0. ②Generation of the masked-input for the encoder in inClust+. ③Data encoding, covariates elimination and data integration. ④Reconstruction of expression profile for both common genes and scRNA-seq-specific genes. ⑤Generation of the masked-output for loss calculation. ⑥Calculation of the loss for backpropagation. Imputation: after training, the output of the decoder (step ④) would impute the missing scRNA-seq-specific genes in MERFISH data. **B** Training inClust+ with scRNA-seq data. In encoder, only the expression data of common genes are the effective inputs. So, in the first layer of the encoder, only the corresponding connections actually contribute to the encoding process. In decoder, both common genes and scRNA-seq-specific genes are reconstructed and pass through the mask. The loss between input and output with both the common and specific genes is calculated, and all connections in the last layer contribute to the loss. In short, inClust+ uses common genes to reconstruct common genes and scRNA-seq-specific genes. **C** Training inClust+ with MERFISH data. In encoder, only the expression data of common genes are the effective inputs. So, in the first layer of the encoder, only the corresponding connections actually contribute to the encoding process. In decoder, both common gene and scRNA-seq-specific gene are reconstructed, while the scRNA-seq-specific genes are filtered out by the output-mask. Loss is calculated according to the common genes, so only connections corresponding to common genes in the last layer of decoder contribute to the calculation of loss. In short, inClust+ uses common genes to reconstruct common genes. However, after training, inClust+ would output common genes and scRNA-seq-specific genes from the input of common genes

The rationale for integrating scRNA-seq and MERFISH data by inClust+ is simple: just treat the multimodal data from different modalities as scRNA-seq data from different batches. In addition, the input-mask module and the output-mask module would enable the gene imputation in MERFISH data based on transferring knowledge from scRNA-seq data (Fig. 2A). For scRNA-seq data, inClust+ uses the common genes to reconstruct common genes and scRNA-seq-specific genes. The reconstructed expression profile of the common genes and the scRNA-seq-specific genes were compared with the real expression profile to update the model parameters (Fig. 2B). For MERFISH data, only reconstructed expression profile of common genes is used for parameter updating (Fig. 2C). Although expression of scRNA-seq-specific genes in MERFISH data did not contribute to the updating of model parameters, they were still reconstructed as a by-product. Since the encoder and decoder used for scRNA-seq and MERFISH data are the same, the reconstructed expression of scRNA-seq-specific genes in MERFISH data could depend on the knowledge transferred from scRNA-seq data (Fig. 2A).

**Fig. 3:**
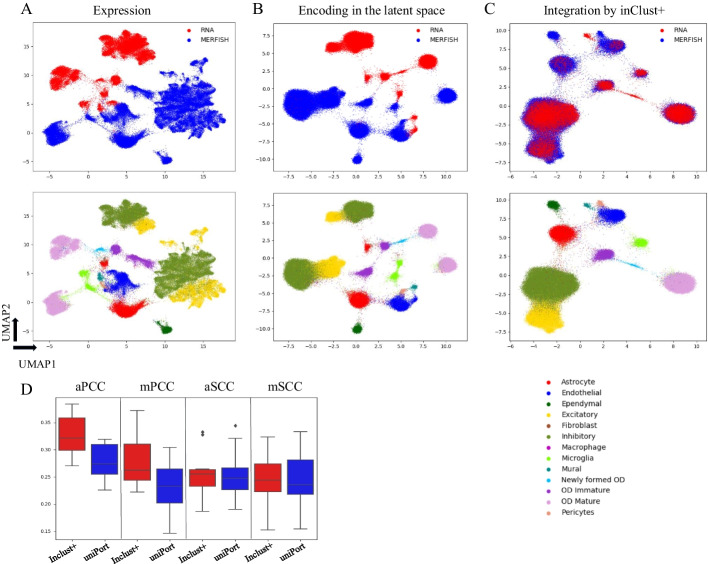
The results for integration of multiple monomodal (unpaired) data and subsequently gene imputation by inClust+. **A** The UMAP plot of the scRNA-seq and MERFISH data (the top 50 PCs) colored by the modalities (top) and cell types (bottom). **B** The UMAP plot of the low dimensional representations with covariate effects for the scRNA-seq and MERFISH data in inClust+ colored by the modalities (top) and cell types (bottom). **C** The UMAP plot of the low dimensional representations without the covariate effects for the scRNA-seq and MERFISH data in inClust+ colored by the modalities (top) and cell types (bottom). **D** Comparison of imputation capability of inClust+ and uniPort. Boxplots of aPCC and mPCC (n = 12), and aSCC and mSCC (n = 12) between real and imputed MERFISH genes generated by inClust+ and uniPort were plotted

For comparison, we randomly selected 80% of genes in MERFISH data as common genes, and the rest as test genes (scRNA-seq-specific) waiting for imputations, as described in the uniPort [18]. The inClust+ first encodes the scRNA-seq and MERFISH data into latent space respectively. As the input data (Fig. 3A), the encoded representations from different modalities are also separated in the latent space (Fig. 3B). After covariates (modalities) removal by vector subtraction, the samples from different modalities were mixed together and clustered according to their cell types (Fig. 3C). As the uniPort [18], the evaluation of imputation is calculated using median and average Spearman correlation coefficients (mSCC and aSCC), and the median and average Pearson correlation coefficients (mPCC and aPCC) over imputed and true testing genes. As shown in the plot, inClust+ demonstrated the higher mSCC (0.243), aSCC (0.255), mPCC (0.263), and aPCC (0.322), above those of uniPort (mSCC of 0.236, aSCC of 0.247, mPCC of 0.233, and aPCC of 0.274) (Fig. 3D).

### InClust+ integrate multi-omics datasets

**Fig. 4:**
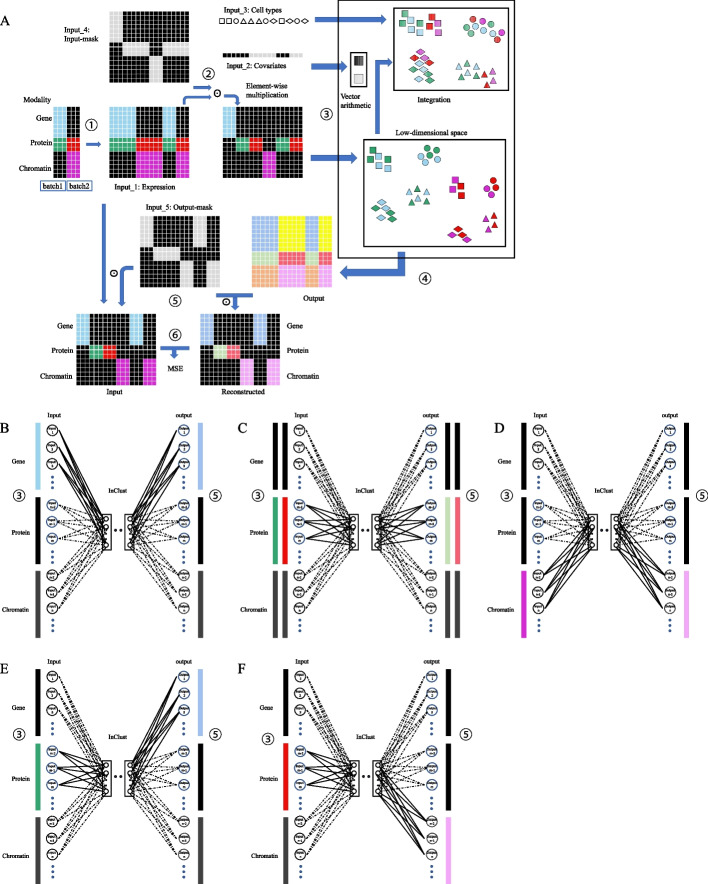
The diagram for integration of multimodal (triple) datasets by inClust+ (see details in Additional file 2). **A** The workflow. Training: ①Generation of the training dataset (Blue: gene expression from dataset 1; Green and red: protein abundance from dataset 1 and dataset 2; Purple: chromatin accessibility from dataset 2; Black: 0-value padding). ②Generation of the masked-input for the encoder in inClust+. ③Data encoding, covariates elimination and data integration. ④Reconstruction for data in all three modalities (Dark blue and yellow: reconstructed gene expression for dataset 1 and dataset 2; Light green and light red: reconstructed protein abundance for dataset 1 and dataset 2; Orange and Light purple: reconstructed chromatin accessibility for dataset 1 and dataset 2). ⑤Generation of the masked-output for loss calculation. ⑥Calculation of the loss for backpropagation. Data integration: after training, encoded low-dimensional representations are mixed together and clustered according to the cell types without the effect of covariate (batches and modalities). **B**–**D** Self-reconstruction. **B** In the first training phase, only gene expression data is effective for input (Blue long strip) and output (Dark blue long strip). Therefore, only the corresponding connections in the first layer (upper part) of the encoder and the last layer (upper part) of the decoder actually contribute to the training process. In short, inClust+ uses gene expression data to reconstruct itself. **C** In the second and third training phases, only protein abundance data is effective for input (Green and red long strip) and output (Light green and light red long strip). Therefore, only the corresponding connections in the first layer (middle part) of the encoder and the last layer (middle part) of the decoder actually contribute to the training process. In short, inClust+ uses protein abundance data to reconstruct itself. **D** In the fourth training phase, only chromatin accessibility data is effective for input (Purple long strip) and output (Light purple long strip). Therefore, only the corresponding connections in the first layer (lower part) of the encoder and the last layer (lower part) of the decoder actually contribute to the training process. In short, inClust+ uses chromatin accessibility data to reconstruct itself. **E**, **F** alternative-reconstruction. **E** In the fifth training phase, only protein abundance data is effective for input (Green long strip) and gene expression data is effective for output (Dark blue long strip). Therefore, only the corresponding connections in the first layer (middle part) of the encoder and the last layer (upper part) of the decoder actually contribute to the training process. In short, inClust+ uses protein abundance data to reconstruct gene expression data. **F** In the sixth training phase, only protein abundance data is effective for input (Red long strip) and chromatin accessibility data is effective for output (Light purple long strip). Therefore, only the corresponding connections in the first layer (middle part) of the encoder and the last layer (lower part) of the decoder actually contribute to the training process. In short, inClust+ uses protein abundance data to reconstruct chromatin accessibility data

Single cell multi-omics can extract information from different cellular components of a single cell at the same time, with different data types and lengths. By flexibly adjusting the input (output) mask modules, inClust+ can be transformed into a model specially used for multimodal data processing. In order to use data from multiple modalities at the same time, data from all modalities are stacked together in the input (Fig. 4A). Accordingly in the model, the first layer of encoder could be regarded as multiple independent neural network layers stacked together, and each part corresponds to data from one modality (Fig. 4B–F) (e.g. one for gene expression, one for protein abundance, and one for chromatin accessibility). The last layer of the decoder is also divided into multiple parts, respectively, with each part reconstructing the data from one modality. The model training is divided into multiple stages, which could be grouped as self-reconstruction and alternative-reconstruction. The self-reconstruction means inClust+ uses data from one modality to reconstruct itself (e.g. in Fig. 4B, inClust+ uses the gene expression data to reconstruct gene expression data). On the contrary, alternative-reconstruction means inClust+ uses data from one modality to reconstruct data from another modality (e.g. in Fig. 4E, inClust+ uses the protein abundance data to reconstruct gene expression data). The rationale is as follows: in the stages of self-reconstruction, each component of the first layer of the encoder is coupled with the corresponding part of the last layer of the decoder. This could be thought of as an encoder/decoder combination for data from one modality. Each encoder/decoder combination updated themselves relatively independently. In contrast, in the stage of alternative-reconstruction, each component of the first layer of the encoder is coupled with the alternative part of the last layer of the decoder. This is an attempt to translate between different modalities in a single cell and integrate them more thoroughly. Furthermore, the batch effect between different datasets could be explicitly removed by vector arithmetic in the latent space as the original inClust (Fig. 4A).

We first applied inClust+ to integrate the multimodal PBMC data with scATAC-seq data and scRNA-seq data (Additional file 1: Fig S1). Before integration, scATAC-seq data and scRNA-seq data were separated in the original space (Additional file 1: Fig S2A). After integration by inClust+, the data from scATAC-seq and scRNA-seq are mixed together in the latent space (Additional file 1: Fig S2B). As in uniPort, the Batch Entropy score is used to measure the degree of mixing cells across datasets and the Silhouette coefficient is used to evaluate the separation of biological distinctions [18]. The result shows that inClust+ has obtained a Batch Entropy score of 0.686 and a Silhouette coefficient of 0.808, which is much higher than those of uniPort, harmony and scVI (Batch Entropy score of 0.619, 0.678 and 0.576. Silhouette coefficient of 0.64, 0.604 and 0.616) (Additional file 1: Fig S2C).

**Fig. 5:**
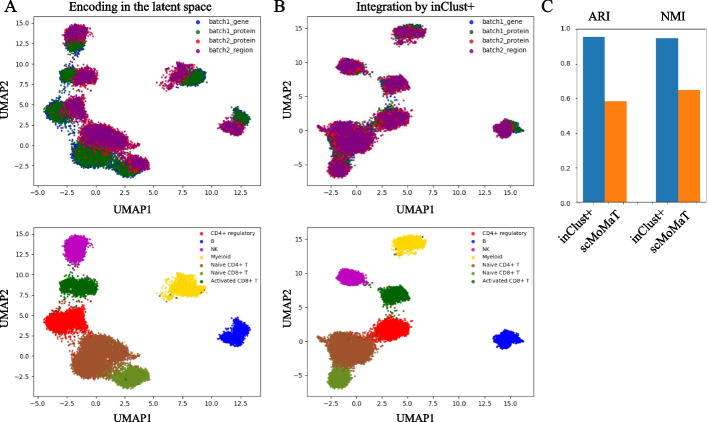
The results for integration of multiple multimodal datasets by inClust+. **A** The UMAP plot of low dimensional representations with batch effects for CITE-seq and ASAP-seq data in inClust+ colored by the covariate (top) and cell types (bottom). **B** The UMAP plot of low dimensional representations without batch effects for CITE-seq and ASAP-seq data in inClust+ colored by the covariate (top) and cell types (bottom). **C** Comparison of data integration results of inClust+ and scMoMaT. Barplots of ARI and NMI for the results of inClust+ and scMoMaT were plotted

We then applied our model to integrate multiple multimodal datasets with batch effect. In the first example, two CITE-seq datasets from different donors with batch effects are used (Additional file 1: Fig S3). Both gene expression data (Additional file 1: Fig S4A) and protein abundance data (Additional file 1: Fig S4B) in CITE-seq datasets have batch effects. The inClust+ integrates data from different modalities in the latent space (Additional file 1: Fig S4C). And the vector arithmetic further integrates data from different batches (Additional file 1: Fig S4D). The result shows that inClust+ has obtained a Batch Entropy score of 0.641 and a Silhouette coefficient of 0.724, which is much higher than those of harmony and scVI (Batch Entropy score of 0.225 and 0.375, Silhouette coefficient of 0.416 and 0.39) (Additional file 1: Fig S4E). In the second example, a CITE-seq dataset (gene expression and protein abundance) and an ASAP-seq dataset (protein abundance and chromatin accessibility) are used (Fig. 4) [27]. There are three modalities (gene expression, protein abundance, chromatin accessibility), where protein abundance data exist in both datasets with batch effect. As in the first example, inClust+ integrates data from different modalities in the latent space (Fig. 5A). And the vector arithmetic further integrates data from different batches (Fig. 5B). We compared the integration results of inClust+ with that of scMoMat through the metrics of adjusted rand index (ARI) and the normalized mutual information (NMI), which used the previous identified seven cell type labels as the ground truth clustering labels [27]. The result shows that inClust+ has obtained an ARI of 0.957 and an NMI of 0.949, which is much higher than those of scMoMat (ARI of 0.585 and NMI of 0.650) (Fig. 5C).

### Cross-modal generation by inClust+ 

**Fig. 6:**
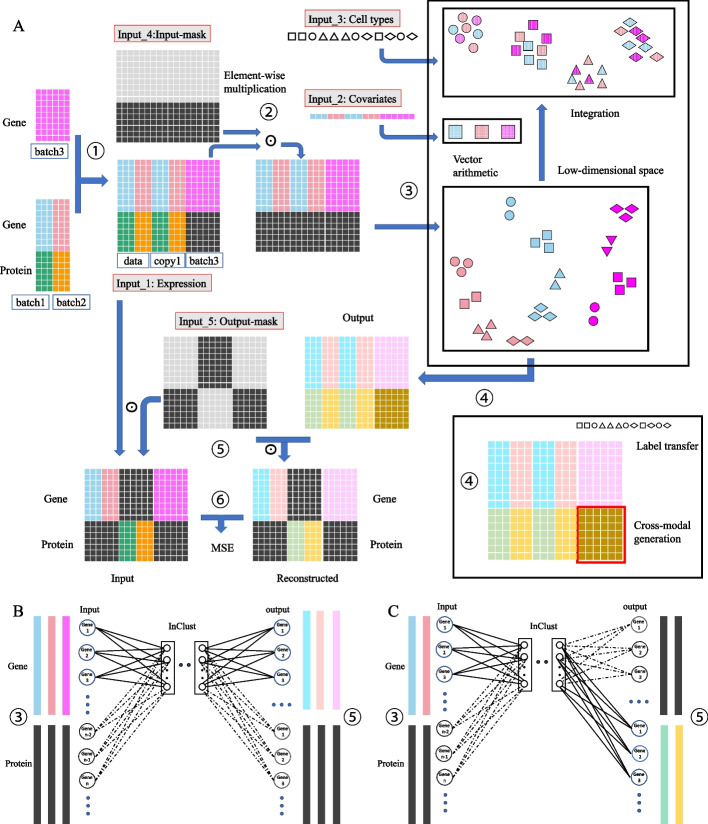
The diagram for cross-modal generation of inClust+ (see details in Additional file 2). **A** The workflow. Training: ①Generation of the training dataset (Blue, red and purple: gene expression from dataset 1, dataset 2 and dataset 3; Green and orange: protein abundance from dataset 1 and dataset 2; Black: 0-value padding).. ②Generation of the masked-input for the encoder in inClust+. ③Data encoding, covariates elimination and data integration. ④The decoder simultaneously outputs the reconstructed gene expression data and the reconstructed protein abundance data (Light blue, light red and light purple: reconstructed gene expression for dataset 1, dataset 2 and dataset 3; Light green, faint yellow and brown: reconstructed protein abundance for dataset 1, dataset 2 and dataset 3). ⑤Generation of the masked-output for loss calculation. ⑥Calculation of the loss for backpropagation. Label transfer and cross-modal generation: after training, the labels are transferred from cells of multimodal data to the cells of monomodal data in the same clusters. The output of the decoder (step ④) would generate the missing modality in monomodal data. **B** Training inClust+ with gene expression data. In these stages, only gene expression data is effective for input (Blue, red and purple long strip) and output (Light blue, light red and light purple long strip). Therefore, only the corresponding connections in the first layer (upper part) of the encoder and the last layer (upper part) of the decoder actually contribute to the training process. In short, inClust+ uses gene expression data to reconstruct itself. **C** Training inClust+ with gene expression data and translating them into protein abundance data. In these stages, only gene expression data is effective for input (Blue and red long strip) and protein abundance data is effective for output (Light green and faint yellow long strip). Therefore, only the corresponding connections in the first layer (upper part) of the encoder and the last layer (lower part) of the decoder actually contribute to the training process. In short, inClust+ uses gene expression data to reconstruct protein abundance data

The multi-omics dataset contains data from multiple modalities, and could be used as a reference to complete the monomodal data into multimodal data. Our inClust+ can extract information from multi-omics reference, and translate monomodal data into data of another modality. As the situation for multimodal integration, the first layer of encoder and the last layer of decoder could be regarded as multiple independent neural network layers stacked together to handle the stack data of multiple modalities (Fig. 6A). The translation from data of gene expression into data of protein abundance in the multimodal reference was carried out in two stages in each round of training. In the first stage, inClust+ uses the gene expression data to reconstruct itself (Fig. 6B). Alternatively, in the second phase, inClust+ uses the gene expression data to reconstruct protein abundance (Fig. 6C). There is the third stage for the monomodal data that needs to be completed. In this stage, inClust+ uses the gene expression data to reconstruct itself in the monomodal dataset (Fig. 6B). After training, inClust+ could transfer labels from the gene expression data in the multimodal reference to the gene expression data in the monomodal dataset. Meanwhile, based on the gene expression data in the monomodal dataset, the corresponding protein abundance data could be generated by automatic translation.

**Fig. 7:**
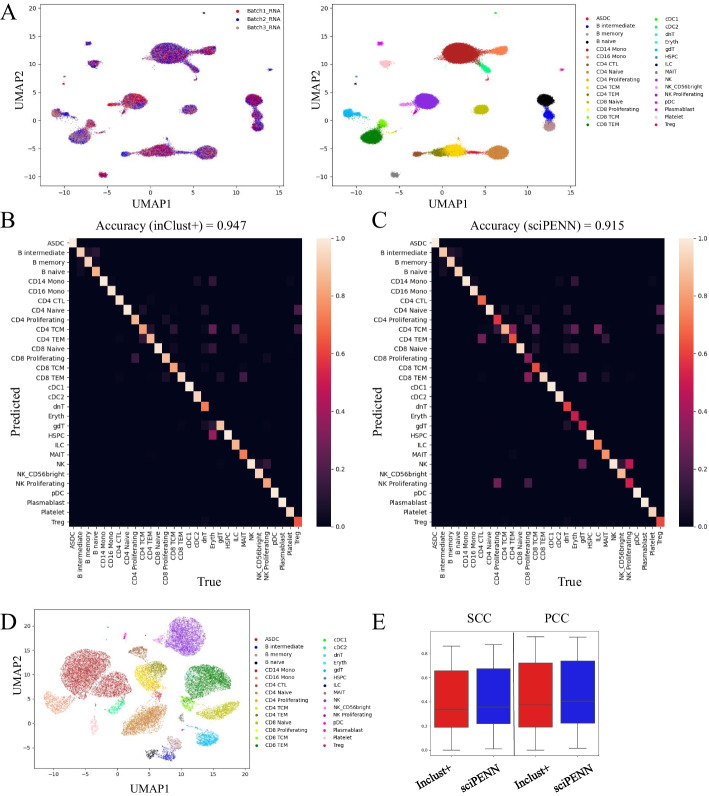
The results for cross-modal generation by inClust+. **A** The UMAP plot of the low dimensional representations without the covariate effects for the gene expression data in inClust+ colored by the covariate (left) and cell types (right). **B** Heatmap for the confusion matrix of results generated by inClust+ with average accuracy above. **C** Heatmap for the confusion matrix of results generated by sciPENN with average accuracy above. **D** The UMAP plot visualization of generated protein abundance data by inClust+. **E** Comparison of cross-modal generation results of inClust+ and sciPENN. Boxplots of PCC, and SCC between real and generated Protein produced by inClust+ and sciPENN were plotted

We evaluated the capability of inClust+ to complete a monomodal dataset into a multimodal dataset through two CITE-seq references and a scRNA-seq dataset. The UMAP plots show that inClust+ could integrate the gene expression data from different datasets well (Fig. 7A, Additional file 1: Fig S5). And the results of the labels transferring are plotted in the confusion matrix, which show that inClust+ is better than sciPENN, with the accuracies of 0.947 in inClust+ (Fig. 7B) and 0.915 in sciPENN (Fig. 7C). The generated protein abundance data by inClust+ was visualized by UMAP (Fig. 7D). And the prediction accuracy of protein abundance was measured by calculating the Pearson correlation and the Spearman correlation between the predicted data and real data. The results show that inClust+ (mSCC of 0.334, mPCC of 0.376) is comparable to sciPENN (mSCC of 0.356, mPCC of 0.405), which is specially optimized for protein abundance prediction related to CITE-seq multimodal data [25] (Fig. 7E).

## Discussion

In this paper, we described a means to enhance inClust through adding an input-mask module and an output-mask module, and called the augmented version of model inClust+. We applied inClust+ to various datasets, ranging from multiple monomodal (unpaired) datasets, one or several multimodal datasets, and datasets containing multimodal data and monomodal data. In these examples, inClust+ demonstrated its capability of data integration, imputation and data generation. Firstly, through the merits of mask modules, inClust+ was used to impute MERFISH data by referring to scRNA-seq data with similar cell population. Then, the capability of inClust+ with stacked encoder-decoder architecture and mask modules for multimodal integration was evaluated on three examples. The results show that inClust+ can’t only mix data between modalities, but also separate biological differences and remove the batch effect. Finally, inClust+ was used to integrate data with both monomodal dataset and multimodal dataset. The results show that inClust+ can transfer labels from multimodal data to monomodal data, and complete the missing modality in monomodal data. The application of inClust+ is not limited to the above cases. For gene imputation, there will be a situation where all datasets have their own specific genes, rather than just one dataset with its own unique genes. By adjusting the output mask, inClust+ can integrate the two datasets based on the shared genes, and impute the rest genes in both datasets by referring to the specific genes in the corresponding dataset. For missing modality generation, there will be a situation where all datasets have their own specific modalities, inClust+ can integrate both datasets based on the shared modalities and generate the missing modality in each dataset by referring to the specific modality in the corresponding dataset.

Because inClust+ is an extension of inClust in multimodal applications, inClust+ and inClust can be put together as a whole when compared with other integration methods. What distinguishes our model (inClust and inClust +) from other integration methods lies in its flexibility to adapt to different situations and its ability to integrate information as much as possible. The flexibility is reflected in the following two points. Firstly, as we described in inClust, the label information could be flexibly handled [26]. This merit is also inherited by inClust+, and is reflected in the fact that inClust+ can transfer labels from reference dataset to query dataset in semi-supervised mode. Secondly, the two mask modules in inClust+ could be flexibly adjusted to deal with different inputs. The model's ability to integrate information as much as possible is embodied in the following two points. Firstly, it is proved in inClust that the model could use not only expression data, but also covariant information (e.g. batch) and label information [26]. This merit is also inherited by inClust+. Secondly, as shown in inClust+, the model could utilize not only the shared data (shared gene expression or shared modality) to integration, but also specific genes or modality to missing genes imputation or missing modality generation. In short, our model can not only integrate data, but also complete other downstream tasks on the basis of data integration (e. g. Out-of-distribution generation, label transfer and new type identification, spatial domain segmentation, cross modal imputation and generation).

Adding masks is a common way to enhance models in deep learning [28]. In inClust+, we augment our model through a pair of mask modules (the input-mask module and the output-mask module). The flexible design and use of masks enable model to complete a series of tasks, which usually need to be completed by multiple models respectively. For example, inClust+ can utilize the common and dataset-specific genes for integration and imputation, as uniPort [18]. Masking makes things simple: the input-mask screens out common genes and the output-mask screens out common and dataset-specific genes of the corresponding data. Meanwhile, inClust+ could integrate multimodal dataset to achieve multi-domain translation, as cross-modal autoencoder [21]. Input-mask and output-mask make inClust+ into multiple independent and related encoder-decoder combinations. Therefore, inClust+ can not only compress and reconstruct the data from the same modality, but also compress the data from one modality and reconstruct it into another modality, thus realizing cross-modal translation. Furthermore, inClust+ could integrate multimodal datasets and monomodal dataset, transfer labels from multimodal data to monomodal data, and complete monomodal data into multimodal data by data generation, as sciPENN [25]. InClust+ refers to multimodal dataset to generate the data of missing modality in monomodal dataset. Generally speaking, as a model augmentation technology, adding a pair of masks to the model is not only limited to inClust, but also can be extended to deep learning models with similar encoder-decoder structures, such as scArches [29].

## Conclusions

The inClust+ gains the ability to process multimodal data by using two mask modules. It could impute genes in MERFISH data by referring scRNA-seq data with similar cell populations. It was also shown to have the capability to integrate the multimodal data (e.g. tri-modal data with gene expression, chromatin accessibility and protein abundance) with batch effect. Furthermore, inClust+ was used to integrate an unlabeled monomodal scRNA-seq dataset and labeled multimodal CITE-seq datasets, transfer label from CITE-seq datasets to scRNA-seq dataset, and generate the missing modality of protein abundance in monomodal scRNA-seq data. Although the tasks mentioned above are different, inClust+ can flexibly change the mask modules to adapt to the corresponding tasks. And the performance of inClust+ in the corresponding tasks is better than or comparable to the latest tools. The successful implementation of mask in inClust+ implies that the augmentation through mask modules has application in other deep learning methods with similar encoder-decoder architecture to broaden the application scope of these models.

## Methods

### Datasets and preprocessing

Brain scRNA-seq and MERFISH dataset: The mouse brains' scRNA-seq and spatial transcriptomics datasets were obtained from Gene Expression Omnibus (GSE113576) [30] and Dryad repositories [https://datadryad.org/stash/dataset/10.5061/dryad.8t8s248], respectively. Then, the data were preprocessed according to the method of Cao et al. [18]. We obtained 30,370 cells in scRNA-seq and 64,373 cells in MERFISH with 153 common genes.

Human PBMC paired multi-omics dataset: The single cell paired-omics dataset including DNA accessibility and gene expression comes from the publicly available dataset (PBMCs from C57BL/6 mice (v1, 150 × 150), Single Cell Immune Profiling Dataset by Cell Ranger 3.1.0. 10 × Genomics, 2019), and were preprocessed according to the method of Cao et al. [18]. We obtained 11,259 cells with 2000 highly variable common genes across cells of all datasets.

Human PBMC dataset (soMoMat): The CITE-seq and ASAP-seq human PBMC dataset is available at Gene Expression Omnibus under accession number GSE156478 [31] and the dataset after filtering is available at https://github.com/PeterZZQ/scMoMaT [27]. We preprocessed the dataset using the method of Zhang et al. [27] and we selected 2 batches of these cells which batch1 (5023 cells) is measured with gene expression and protein abundance simultaneously using CITE-seq and batch2 (3517 cells) is measured with protein abundance and chromatin accessibility simultaneously using ASAP-seq. The integrating three-modality matrices of the human PBMC dataset contain overlapping 4768 genes, 17,442 regions and 216 proteins.

Human PBMC and MALT CITE-seq dataset: The CITE-seq of human PBMC datasets was obtained from Gene Expression Omnibus (GSE164378) [32]. Then, we selected highly variable genes (HVGs) according to the method of Lakkis et al. [25]. For gene, we used the scanpy 1.7.1 to normalize expression value [33]. Finally, for the PBMC dataset, we obtained 161,748 cells with 1000 HVGs and 224 proteins. Cells from donor 7 (25,827 cells) and donor 8 (26,208 cells) were used in the integration experiment. In cross-modal generation experiment, cells from donor 6 (20,651 cells) and donor 7 were used as multimodal dataset and scRNA-seq data in donor 8 was used as monomodal dataset.

### InClust+ : overview

InClust+ is based on inClust applied for transcriptome data [26]. InClust+ is the multi-modal version of inClust, with an input mask module in front of the encoder and an output module after the encoder (Fig. 1A).

### Architecture of inClust+ 

#### Input

InClust+ take 5 inputs, input 1 is the multimodal data and input 2 is the covariates information such as batch or modality. Input 3 is the label information, which is optional. Input 4 is the input-mask for screening out input. Input 5 is the output-mask for screening out output.

#### Input-mask module

The input-mask is a matrix as big as the input, with 0 or 1 in each element. The input is multiplied element-wise with the input-mask matrix to screen out the desired elements.

#### Encoder

The encoder is a three-layer neural network with a non-linear function as the activation function.

#### Latent sampling layer

A neural network without activation function is used to estimate mean (μ_z_) and standard deviation (Σ_z_). The reparameterization trick was used for sampling latent variables Z_1_.

#### Embedding layer

The embedding layer embeds the auxiliary information (input2) into the latent space as a real-valued vector.

#### Vector arithmetic layer

The vector arithmetic is performed in the latent space. The estimated mean (μ_z_) would substrate (or add) the embedding vector E. The resulting vector Z_2_ retains the real biological information after removing the unwanted covariates or mixing the auxiliary information.

#### Classifier

The real-valued vector Z_2_ will pass through a neural network with softmax as the activation function. The output of the classifier is the output2.

#### Decoder

The decoder is a three-layer neural network with non-linear function as the activation function.

#### Output-mask module

The output-mask is a matrix as big as the output, with 0 or 1 in each element. The output is multiplied element-wise with the output-mask matrix to screen out the desired elements.

### Pseudocode

1. screen out the input by masking: input_in_ = input_1_⊙ input_4_(input-mask)
2. encode the input into latent space: h_2_ = relu(W_2_(relu(W_1_* input_in_)))
3. estimate the parameter: μ_z_ = W_μ_h_2_, Σ_z_ = W_Σ_h_2_
  Reconstruction:
4. reparameterization trick: Z_1_ = μ_z_ + Σ_z_*εε ~ N(0,1)
5. decode the latent space vector z into expression profile:
  output_1_ = relu(W_out_(relu(W_3_Z_1_)))
6. screen out the output by masking: output = output_1_⊙input_5_(output-mask)
  Clustering:
7. embed the auxiliary information: *E* = W_E_*input_2_
8. latent space vector arithmetic: Z_2_ = μ_z_ − *E* (or μ_z_ + *E*)
9. classification (clustering): output_2_ = softmax(W_C_Z_2_)

### Methods for comparison

#### uniPort

UniPort from the python package uniPort to the comparison of data integration and imputation [18].

#### Harmony

Harmony from the R package harmony to the comparison of data integration [34].

#### scVI

ScVI from scvi-tool to the comparison of data integration [35].

#### scMoMaT

ScMoMaT from the python package scMoMaT to the comparison of data integration [27].

#### sciPENN

SciPENN from the python package sciPENN to the comparison of cross-modal generation [25].

### Supplementary Information

**Additional file 1**. Supplementary Figures.

**Additional file 2**. Additional Notes on Figures 2, 4 and 6.

## Data Availability

InClust+ model is open source, and available for download from https://github.com/wanglf19/inClust_plus. It is implemented in python 3.6.

## Abbreviations

scRNA-seq: Single cell RNA sequencing
scATAC-seq: Single-cell assay for transposase-accessible chromatin sequencing
scBS-seq: Single-cell bisulfite sequencing
CITE-seq: Cellular indexing of Transcriptomes and Epitopes by sequencing
inClust: Integrated clustering
InClust+: Augmented integrated clustering
mSCC: Median Spearman correlation coefficients
aSCC: Average Spearman correlation coefficients
mPCC: Median Pearson correlation coefficients
aPCC: Average Pearson correlation coefficients
ARI: Adjusted rand index
NMI: Normalized mutual information

## References

[1] Tang F, Barbacioru C, Wang Y, Nordman E, Lee C, Xu N, Wang X, Bodeau J, Tuch BB, Siddiqui A (2009). mRNA-Seq whole-transcriptome analysis of a single cell. Nat Methods.

[2] Zheng C, Zheng L, Yoo JK, Guo H, Zhang Y, Guo X, Kang B, Hu R, Huang JY, Zhang Q (2017). Landscape of Infiltrating T Cells in Liver Cancer Revealed by Single-Cell Sequencing. Cell.

[3] Buenrostro JD, Wu B, Litzenburger UM, Ruff D, Gonzales ML, Snyder MP, Chang HY, Greenleaf WJ (2015). Single-cell chromatin accessibility reveals principles of regulatory variation. Nature.

[4] Smallwood SA, Lee HJ, Angermueller C, Krueger F, Saadeh H, Peat J, Andrews SR, Stegle O, Reik W, Kelsey G (2014). Single-cell genome-wide bisulfite sequencing for assessing epigenetic heterogeneity. Nat Methods.

[5] Guo X, Zhang Y, Zheng L, Zheng C, Song J, Zhang Q, Kang B, Liu Z, Jin L, Xing R (2018). Global characterization of T cells in non-small-cell lung cancer by single-cell sequencing. Nat Med.

[6] Saelens W, Cannoodt R, Todorov H, Saeys Y (2019). A comparison of single-cell trajectory inference methods. Nat Biotechnol.

[7] Aibar S, Gonzalez-Blas CB, Moerman T, Huynh-Thu VA, Imrichova H, Hulselmans G, Rambow F, Marine JC, Geurts P, Aerts J (2017). SCENIC: single-cell regulatory network inference and clustering. Nat Methods.

[8] Argelaguet R, Cuomo ASE, Stegle O, Marioni JC (2021). Computational principles and challenges in single-cell data integration. Nat Biotechnol.

[9] Efremova M, Teichmann SA (2020). Computational methods for single-cell omics across modalities. Nat Methods.

[10] Zhu C, Preissl S, Ren B (2020). Single-cell multimodal omics: the power of many. Nat Methods.

[11] Chen S, Lake BB, Zhang K (2019). High-throughput sequencing of the transcriptome and chromatin accessibility in the same cell. Nat Biotechnol.

[12] Cao J, Cusanovich DA, Ramani V, Aghamirzaie D, Pliner HA, Hill AJ, Daza RM, McFaline-Figueroa JL, Packer JS, Christiansen L (2018). Joint profiling of chromatin accessibility and gene expression in thousands of single cells. Science.

[13] Zhu C, Yu M, Huang H, Juric I, Abnousi A, Hu R, Lucero J, Behrens MM, Hu M, Ren B (2019). An ultra high-throughput method for single-cell joint analysis of open chromatin and transcriptome. Nat Struct Mol Biol.

[14] Ma S, Zhang B, LaFave LM, Earl AS, Chiang Z, Hu Y, Ding J, Brack A, Kartha VK, Tay T (2020). Chromatin potential identified by shared single-cell profiling of RNA and chromatin. Cell.

[15] Peterson VM, Zhang KX, Kumar N, Wong J, Li L, Wilson DC, Moore R, McClanahan TK, Sadekova S, Klappenbach JA (2017). Multiplexed quantification of proteins and transcripts in single cells. Nat Biotechnol.

[16] Stoeckius M, Hafemeister C, Stephenson W, Houck-Loomis B, Chattopadhyay PK, Swerdlow H, Satija R, Smibert P (2017). Simultaneous epitope and transcriptome measurement in single cells. Nat Methods.

[17] Clark SJ, Argelaguet R, Kapourani CA, Stubbs TM, Lee HJ, Alda-Catalinas C, Krueger F, Sanguinetti G, Kelsey G, Marioni JC (2018). scNMT-seq enables joint profiling of chromatin accessibility DNA methylation and transcription in single cells. Nat Commun.

[18] Cao K, Gong Q, Hong Y, Wan L (2022). A unified computational framework for single-cell data integration with optimal transport. Nat Commun.

[19] Gong B, Zhou Y, Purdom E (2021). Cobolt: integrative analysis of multimodal single-cell sequencing data. Genome Biol.

[20] Li G, Fu S, Wang S, Zhu C, Duan B, Tang C, Chen X, Chuai G, Wang P, Liu Q (2022). A deep generative model for multi-view profiling of single-cell RNA-seq and ATAC-seq data. Genome Biol.

[21] Yang KD, Belyaeva A, Venkatachalapathy S, Damodaran K, Katcoff A, Radhakrishnan A, Shivashankar GV, Uhler C (2021). Multi-domain translation between single-cell imaging and sequencing data using autoencoders. Nat Commun.

[22] Minoura K, Abe K, Nam H, Nishikawa H, Shimamura T (2021). A mixture-of-experts deep generative model for integrated analysis of single-cell multiomics data. Cell Rep Methods.

[23] Zuo C, Dai H, Chen L (2021). Deep cross-omics cycle attention model for joint analysis of single-cell multi-omics data. Bioinformatics.

[24] Cao ZJ, Gao G (2022). Multi-omics single-cell data integration and regulatory inference with graph-linked embedding. Nat Biotechnol.

[25] Lakkis J, Schroeder A, Su K, Lee MYY, Bashore AC, Reilly MP, Li M (2022). A multi-use deep learning method for CITE-seq and single-cell RNA-seq data integration with cell surface protein prediction and imputation. Nat Mach Intell.

[26] Wang L, Nie R, Zhang Z, Gu W, Wang S, Wang A, Zhang J, Cai J (2023). A deep generative framework with embedded vector arithmetic and classifier for sample generation, label transfer, and clustering of single-cell data. Cell Rep Methods.

[27] Zhang Z, Sun H, Mariappan R, Chen X, Chen X, Jain MS, Efremova M, Teichmann SA, Rajan V, Zhang X (2023). scMoMaT jointly performs single cell mosaic integration and multi-modal bio-marker detection. Nat Commun.

[28] Wang L, Nie R, Zhang J, Cai J (2022). scCapsNet-mask: an updated version of scCapsNet with extended applicability in functional analysis related to scRNA-seq data. BMC Bioinformatics.

[29] Lotfollahi M, Naghipourfar M, Luecken MD, Khajavi M, Büttner M, Wagenstetter M, Avsec Ž, Gayoso A, Yosef N, Interlandi M (2022). Mapping single-cell data to reference atlases by transfer learning. Nat Biotechnol.

[30] Moffitt JR, Bambah-Mukku D, Eichhorn SW, Vaughn E, Shekhar K, Perez JD, Rubinstein ND, Hao J, Regev A, Dulac C (2018). Molecular, spatial, and functional single-cell profiling of the hypothalamic preoptic region. Science.

[31] Mimitou EP, Lareau CA, Chen KY, Zorzetto-Fernandes AL, Hao Y, Takeshima Y, Luo W, Huang TS, Yeung BZ, Papalexi E (2021). Scalable, multimodal profiling of chromatin accessibility, gene expression and protein levels in single cells. Nat Biotechnol.

[32] Hao Y, Hao S, Andersen-Nissen E, Mauck WM, Zheng S, Butler A, Lee MJ, Wilk AJ, Darby C, Zager M (2021). Integrated analysis of multimodal single-cell data. Cell.

[33] Wolf FA, Angerer P, Theis FJ (2018). SCANPY: large-scale single-cell gene expression data analysis. Genome Biol.

[34] Korsunsky I, Millard N, Fan J, Slowikowski K, Zhang F, Wei K, Baglaenko Y, Brenner M, Loh PR, Raychaudhuri S (2019). Fast, sensitive and accurate integration of single-cell data with Harmony. Nat Methods.

[35] Lopez R, Regier J, Cole MB, Jordan MI, Yosef N (2018). Deep generative modeling for single-cell transcriptomics. Nat Methods.

