# Supplementary material for: InClust+: the deep generative framework with mask modules for multimodal data integration, imputation, and cross-modal generation
Source: BMC Bioinformatics. 2024 Jan 24;25:41. doi: 10.1186/s12859-024-05656-2 (PMC10809631; doi:10.1186/s12859-024-05656-2)
Supplement: Supplementary file 1 — Additional file 1. Supplementary Figures. [file 12859_2024_5656_MOESM1_ESM.pdf]

Figure S1

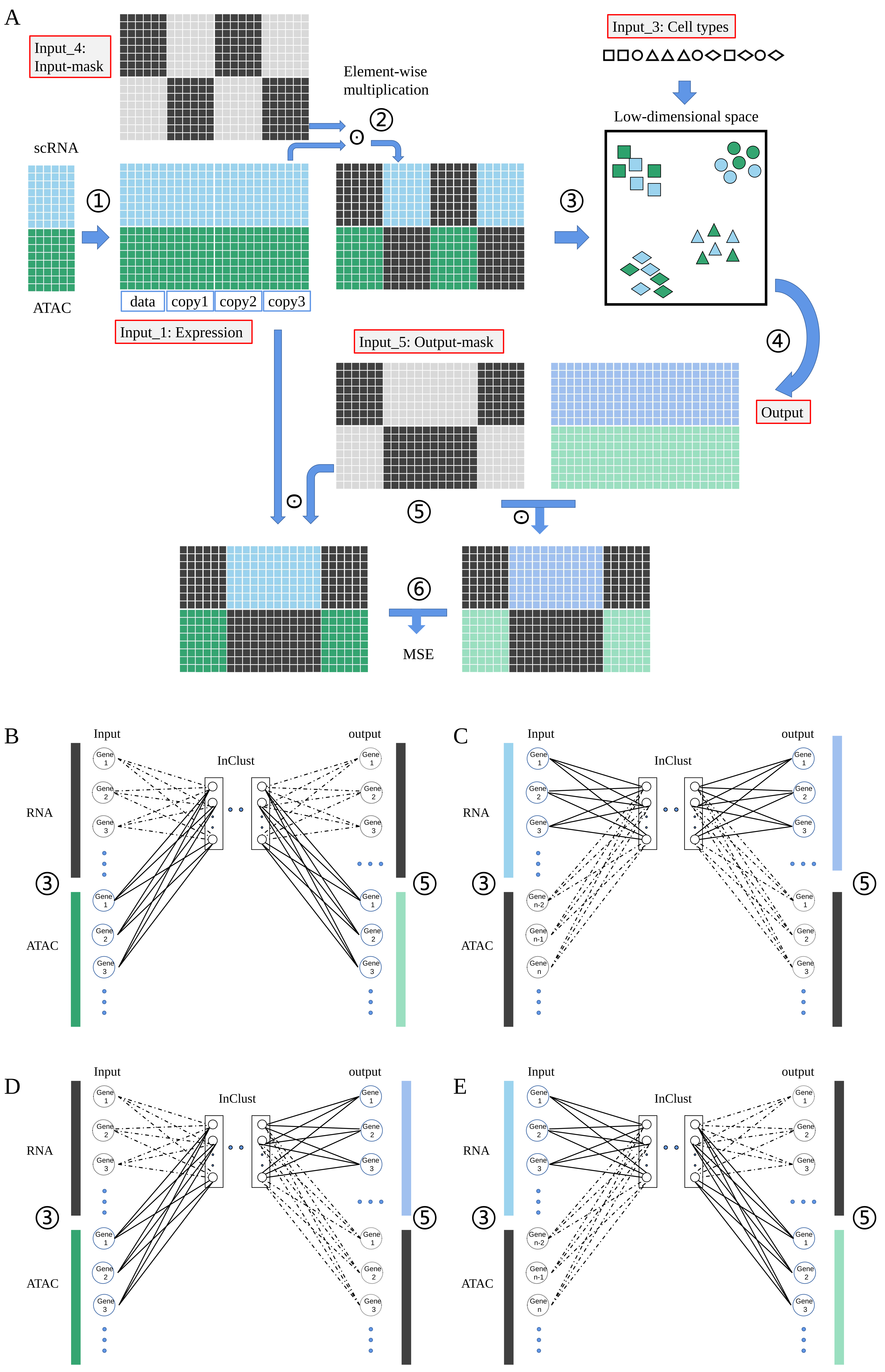

A
Input_4: Input-mask
Element-wise multiplication
②
⊙
Input_3: Cell types
Low-dimensional space
scRNA
①
ATAC
data
copy1
copy2
copy3
Input_1: Expression
③
④
Input_5: Output-mask
Output
⊙
⑤
⊙
⑥
MSE
B
Input
output
InClust
RNA
ATAC
③
⑤
C
Input
output
InClust
RNA
ATAC
③
⑤
D
Input
output
InClust
RNA
ATAC
③
⑤
E
Input
output
InClust
RNA
ATAC
③
⑤

Figure S2

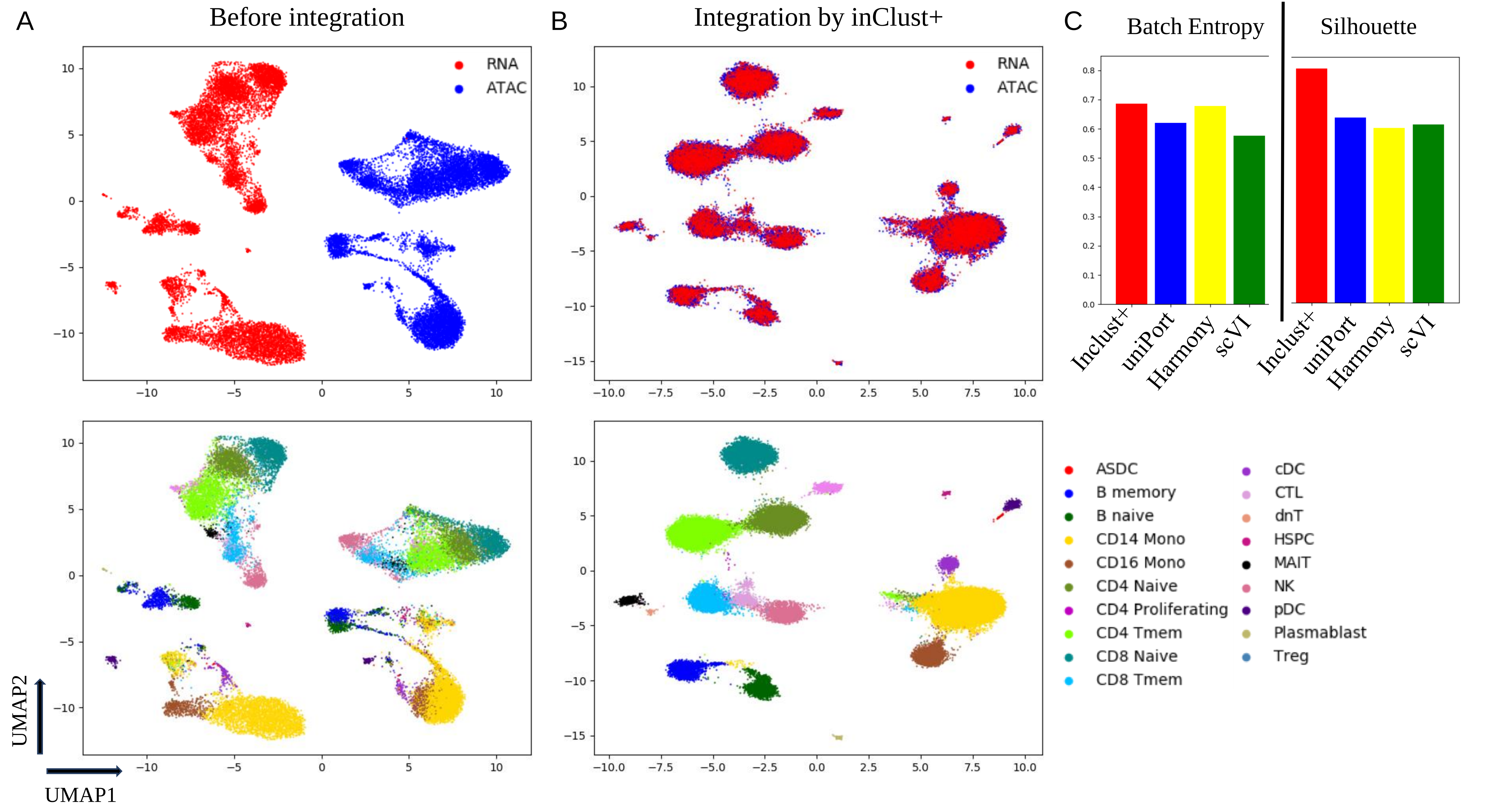
A
Before integration
B
Integration by inClust+
C
Batch Entropy
Silhouette
RNA
ATAC
Inclust+
uniPort
Harmony
scVI
UMAP2
UMAP1
ASDC
B memory
B naive
CD14 Mono
CD16 Mono
CD4 Naive
CD4 Proliferating
CD4 Tmem
CD8 Naive
CD8 Tmem
cDC
CTL
dnT
HSPC
MAIT
NK
pDC
Plasmablast
Treg

Figure S3

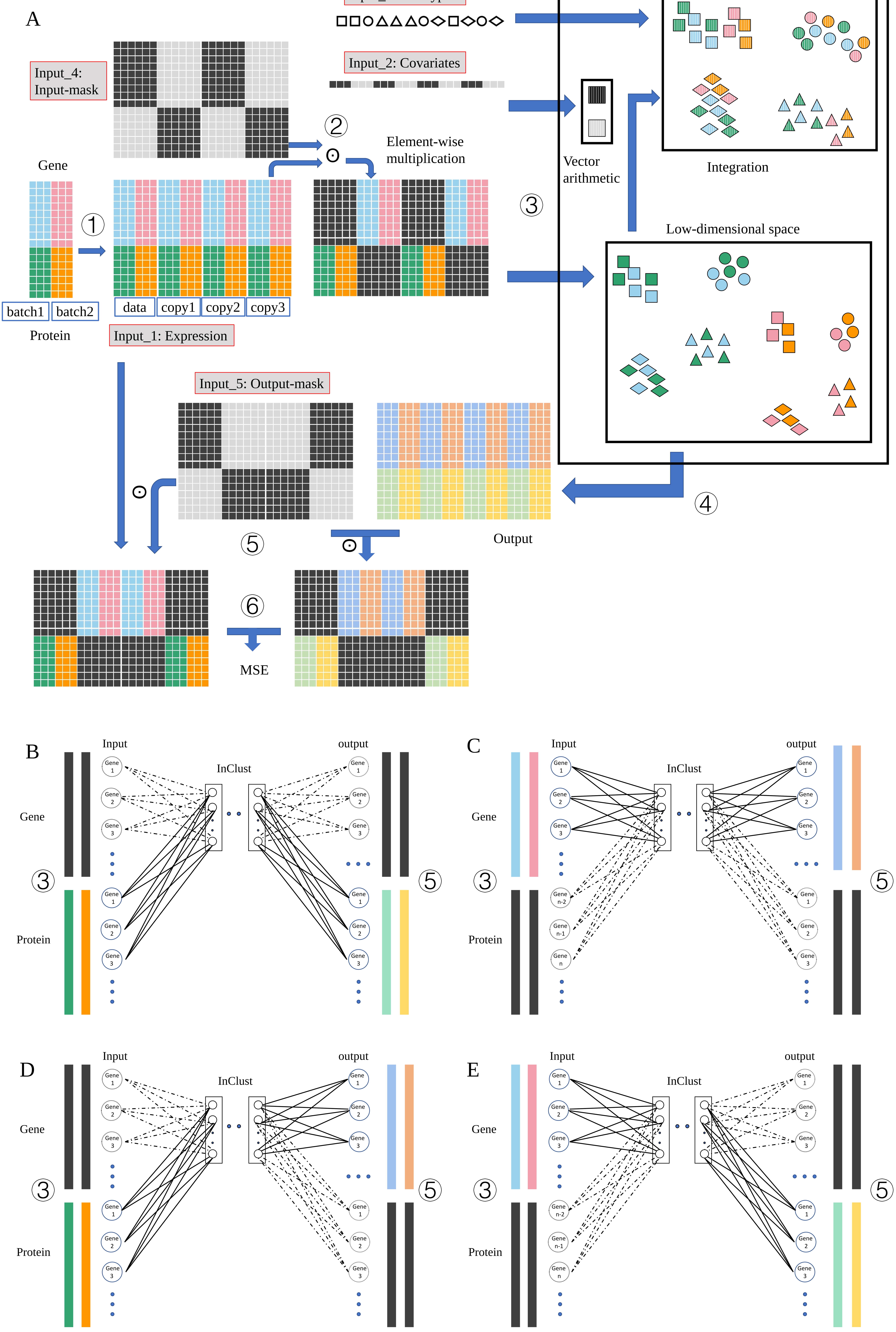

A
Input_4: Input-mask
Input_3: Cell types
Input_2: Covariates
Element-wise multiplication
Vector arithmetic
Integration
Low-dimensional space
Gene
Protein
batch1
batch2
data
copy1
copy2
copy3
Input_1: Expression
Input_5: Output-mask
Output
MSE
B
C
D
E
Input
output
InClust
Gene
Protein

Figure S4

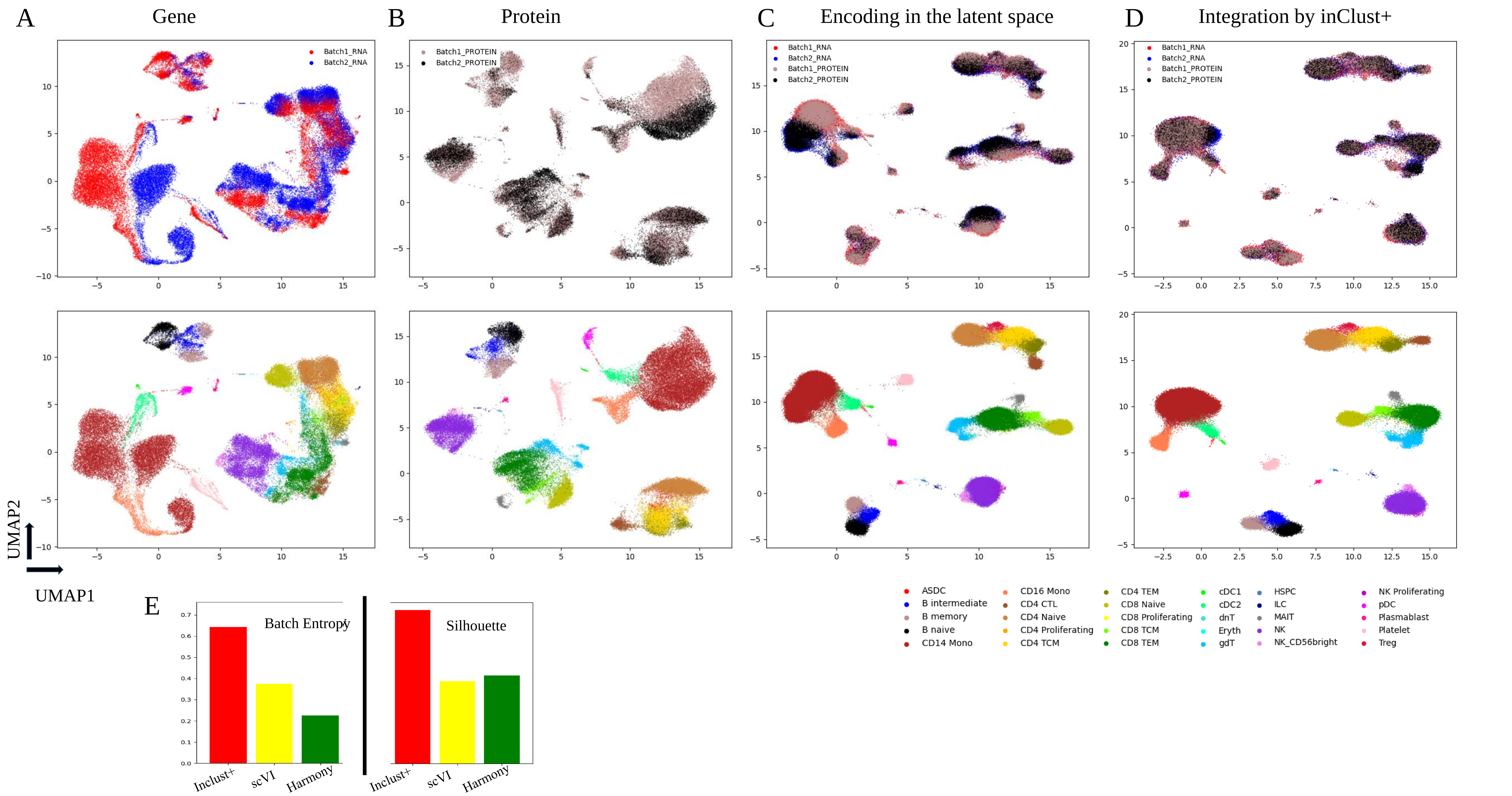
A
Gene
B
Protein
C
Encoding in the latent space
D
Integration by inClust+
Batch1_RNA
Batch2_RNA
Batch1_PROTEIN
Batch2_PROTEIN
UMAP2
UMAP1
E
Batch Entropy
Silhouette
Inclust+
scVI
Harmony
ASDC
B intermediate
B memory
B naive
CD14 Mono
CD16 Mono
CD4 CTL
CD4 Naive
CD4 Proliferating
CD4 TCM
CD4 TEM
CD8 Naive
CD8 Proliferating
CD8 TCM
CD8 TEM
cDC1
cDC2
dnT
Eryth
gdT
HSPC
ILC
MAIT
NK
NK_CD56bright
NK Proliferating
pDC
Plasmablast
Platelet
Treg

Figure S5

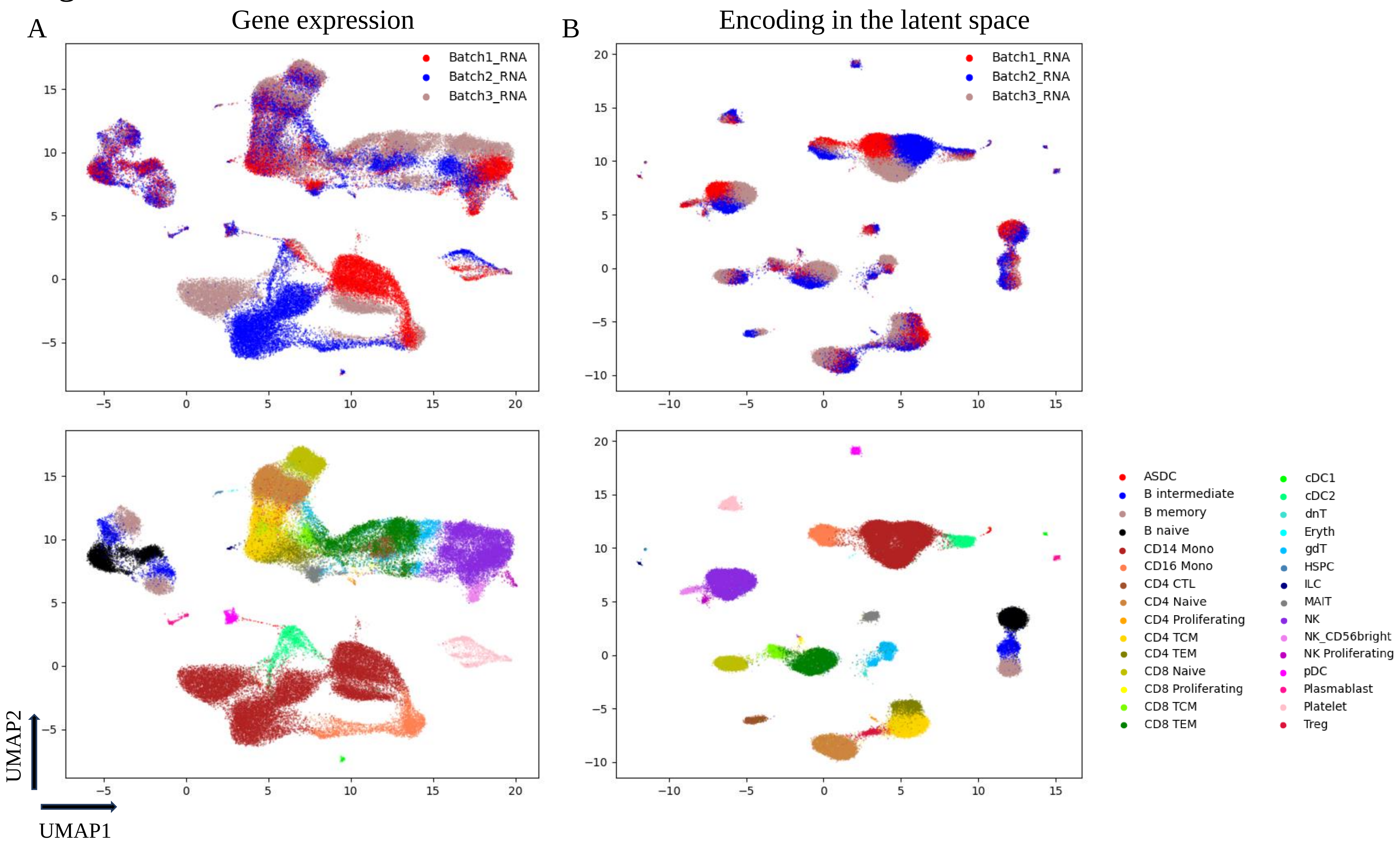
A
Gene expression
B
Encoding in the latent space
Batch1_RNA
Batch2_RNA
Batch3_RNA
UMAP2
UMAP1
ASDC
B intermediate
B memory
B naive
CD14 Mono
CD16 Mono
CD4 CTL
CD4 Naive
CD4 Proliferating
CD4 TCM
CD4 TEM
CD8 Naive
CD8 Proliferating
CD8 TCM
CD8 TEM
cDC1
cDC2
dnT
Eryth
gdT
HSPC
ILC
MAIT
NK
NK_CD56bright
NK Proliferating
pDC
Plasmablast
Platelet
Treg
