# Supplementary material for: InClust+: the deep generative framework with mask modules for multimodal data integration, imputation, and cross-modal generation
Source: BMC Bioinformatics. 2024 Jan 24;25:41. doi: 10.1186/s12859-024-05656-2 (PMC10809631; doi:10.1186/s12859-024-05656-2)
Supplement: Supplementary file 2 — Additional file 2. Additional Notes on Figures 2, 4 and 6. [file 12859_2024_5656_MOESM2_ESM.docx]

**Supplementary Figure legend**

**Figure S1 The diagram for integration of paired PBMC data (scRNA-seq and ATAC-seq) by inClust+**

(**A**) The workflow of inClust+ for integration of paired PBMC data (scRNA-seq and ATAC-seq). Training: ①Generation of the training dataset. To form a training dataset, the data of paired scRNA-seq and ATAC-seq data were duplicated 3 times, and concatenated together. ②Generation of the masked-input for the encoder in inClust. The training dataset multiplies element-wise with an input-mask matrix. The input-mask matrix is as big as the training dataset, and could be equally divided into four parts, each part being as big as the original paired dataset. In the first and the third part, the positions of scRNA-seq data are filled with 0, and the positions of ATAC-seq data are filled with 1. Alternatively, in the second and the fourth part, the positions of scRNA-seq data are filled with 1, and the positions of ATAC-seq data are filled with 0. The result of multiplication is the masked-input for the encoder, with alternate training data of scRNA-seq and ATAC-seq. ③Data encoding and data integration. The data from different modalities (scRNA-seq or ATAC-seq) in the masked-input are encoded by the different parts of encoder into the low-dimensional space, and integrated through the constraints of the cell type information. ④Reconstruction for both scRNA-seq data and ATAC-seq data. The decoder simultaneously outputs the reconstructed scRNA-seq data and the reconstructed ATAC-seq data. ⑤Generation of the mask-output for loss calculation. The output multiplies element-wise with an output-mask matrix. The output-mask matrix is as large as the output, and could be equally divided into four parts, each part being as big as the original paired dataset. In the first and the fourth part, the positions of scRNA-seq data are filled with 0, and the positions of ATAC-seq data are filled with 1. Alternatively, in the second and the third part, the positions of scRNA-seq data are filled with 1, and the positions of ATAC-seq data are filled with 0. The result of multiplication is the masked-output. ⑥Calculation of the loss for backpropagation. The MSE between masked-output and the masked-input is calculated as the loss. Data integration: After training, encoded low-dimensional representations from two modalities (scRNA-seq and ATAC-seq) are mixed together and clustered according to the cell types. (**B**) The first training phase. In this stage, due to the influence of mask, only ATAC-seq data is effective for input and output. Therefore, only the corresponding connections in the first layer (lower part) of the encoder and the last layer (lower part) of the decoder actually contribute to the training process. In short, inClust+ uses ATAC-seq data to reconstruct itself. (**C**) The second training phase. In this stage, due to the influence of mask, only scRNA-seq data is effective for input and output. Therefore, only the corresponding connections in the first layer (upper part) of the encoder and the last layer (upper part) of the decoder actually contribute to the training process. In short, inClust+ uses scRNA-seq data to reconstruct itself. (**D**) The third training phase. In this stage, due to the influence of mask, only ATAC-seq data is effective for input and scRNA-seq data is effective for output. Therefore, only the corresponding connections in the first layer (lower part) of the encoder and the last layer (upper part) of the decoder actually contribute to the training process. In short, inClust+ uses ATAC-seq data to reconstruct scRNA-seq data. (**E**) The fourth training phase. In this stage, due to the influence of mask, only scRNA-seq data is effective for input and ATAC-seq data is effective for output. Therefore, only the corresponding connections in the first layer (upper part) of the encoder and the last layer (lower part) of the decoder actually contribute to the training process. In short, inClust+ uses scRNA-seq data to reconstruct ATAC-seq data.

**Figure S2 The results for integration of multiple multimodal (paired) data by inClust+.**

(**A**) The UMAP plot of the scRNA-seq and ATAC-seq data colored by the covariate (top) and cell types (bottom). (**B**) The UMAP plot of the low dimensional representations without covariate effects for the scRNA-seq and ATAC-seq data in inClust+ colored by the covariate (top) and cell types (bottom). (**C**) The Batch Entropy score and the Silhouette coefficient measure from results of inClust+, uniPort, harmony and scVI.

**Figure S3 The diagram for integration of multimodal (paired) datasets by inClust+**

(**A**) The workflow of inClust+ for integration of multimodal (paired) datasets (gene expression and protein abundance). Training: ①Generation of the training dataset. To form a training dataset, the multimodal (paired) datasets were duplicated 3 times, and concatenated together. ②Generation of the masked-input for the encoder in inClust+. The training dataset multiplies element-wise with an input-mask matrix. The input-mask matrix is as big as the training dataset, and could be equally divided into four parts, each part being as big as the original multimodal (paired) datasets. In the first and the third part, the positions of gene expression data are filled with 0, and the positions of protein abundance data are filled with 1. Alternatively, in the second and the fourth part, the positions of gene expression data are filled with 1, and the positions of protein abundance data are filled with 0. The result of multiplication is the masked-input for the encoder, with alternate training data of gene expression and protein abundance. ③Data encoding, covariates elimination and data integration. The data from different modalities (gene expression or protein abundance) in the masked-input are encoded by the different parts of encoder into the low-dimensional space, and integrated through the constraints of the cell type information. The batch effect is removed by vector subtraction in latent space. ④Reconstruction for both gene expression data and protein abundance data. The decoder simultaneously outputs the reconstructed gene expression data and the reconstructed protein abundance data. ⑤Generation of the masked-output for loss calculation. The output multiplies element-wise with an output-mask matrix. The output-mask matrix is as large as the output, and could be equally divided into four parts, each part being as big as the original multimodal (paired) datasets. In the first and the fourth part, the positions of gene expression data are filled with 0, and the positions of protein abundance data are filled with 1. Alternatively, in the second and the third part, the positions of gene expression data are filled with 1, and the positions of protein abundance data are filled with 0. The result of multiplication is the masked-output. ⑥Calculation of the loss for backpropagation. The MSE between masked-output and the masked-input is calculated as the loss. Data integration: After training, encoded low-dimensional representations are mixed together and clustered according to the cell types without the effect of covariate (batches and modalities). (**B**) The first training phase. In this stage, due to the influence of mask, only protein abundance data is effective for input and output. Therefore, only the corresponding connections in the first layer (lower part) of the encoder and the last layer (lower part) of the decoder actually contribute to the training process. In short, inClust+ uses protein abundance data to reconstruct itself. (**C**) The second training phase. In this stage, due to the influence of mask, only gene expression data is effective for input and output. Therefore, only the corresponding connections in the first layer (upper part) of the encoder and the last layer (upper part) of the decoder actually contribute to the training process. In short, inClust+ uses gene expression data to reconstruct itself. (**D**) The third training phase. In this stage, due to the influence of mask, only protein abundance data is effective for input and gene expression data is effective for output. Therefore, only the corresponding connections in the first layer (lower part) of the encoder and the last layer (upper part) of the decoder actually contribute to the training process. In short, inClust+ uses protein abundance data to reconstruct gene expression data. (**E**) The fourth training phase. In this stage, due to the influence of mask, only gene expression data is effective for input and protein abundance data is effective for output. Therefore, only the corresponding connections in the first layer (upper part) of the encoder and the last layer (lower part) of the decoder actually contribute to the training process. In short, inClust+ uses gene expression data to reconstruct protein abundance data.

**Figure S4 The results for integration of multiple multimodal (paired) datasets by inClust+.**

(**A**) The UMAP plot of the gene expression data colored by the batches (top) and cell types (bottom). (**B**) The UMAP plot of the protein abundance data colored by the batches (top) and cell types (bottom). (**C**) The UMAP plot of the low dimensional representations with batch effects for the gene expression and protein abundance data in inClust+ colored by the covariate (top) and cell types (bottom). (**D**) The UMAP plot of the low dimensional representations without the batch effects for the gene expression and protein abundance data in inClust+ colored by the covariate (top) and cell types (bottom). (**E**) The Batch Entropy score and the Silhouette coefficient measure from results of inClust+, harmony and scVI.

**Figure S5 The results for integration of gene expression data in dataset with multimodal data and monomodal data**

(**A**) The UMAP plot of the gene expression in the dataset colored by the batches (top) and cell types (bottom). (**B**) The UMAP plot of the low dimensional representations with covariate effects for the gene expression data in inClust+ colored by the covariate (top) and cell types (bottom).

**Supplementary explanation to the figure 2, 4 and 6**

Figure 2 The diagram for integration of multiple monomodal (unpaired) data and subsequently gene imputation by inClust+.

(**A**) The workflow of inClust+ for integration of scRNA-seq and MERFISH data, and subsequently gene imputation for MERFISH data. Training: ①Generation of the training dataset. To form a training dataset, the data from scRNA-seq and MERFISH were aligned with common genes, and the missing scRNA-seq-specific genes in MERFISH data were filled with 0. ②Generation of the masked-input for the encoder in inClust+. The training dataset multiplies element-wise with an input-mask matrix. The input-mask matrix is as large as the training dataset. In the input-mask matrix, the positions of common genes are filled with 1, and the positions of scRNA-seq-specific genes are filled with 0. The result of multiplication is the masked-input of the encoder, and only common gene is effective. ③Data encoding, covariates elimination and data integration. Firstly, the expression profiles of common genes are encoded in low-dimensional space, and then the covariates (modalities) are eliminated by subtracting covariates information from expression profile, and the data from different modalities are integrated through the constraints of the cell type information. ④Reconstruction of expression profile for both common genes and scRNA-seq-specific genes. The decoder outputs the expression profile of the common and scRNA-seq-specific genes. ⑤Generation of the masked-output for loss calculation. The output multiplies element-wise with an output-mask matrix, which is as large as the output. In the output-mask matrix, the place of scRNA-seq-specific genes in MERFISH filled with 0 and the other place filled with 1. ⑥Calculation of the loss for backpropagation. The mean squared error (MSE) between masked-outputs and the training data is calculated as the loss. Imputation: After training, the output of the decoder (step ④) would impute the missing scRNA-seq-specific genes in MERFISH data. (**B**) Training inClust+ with scRNA-seq data. In encoder, only the expression data of common genes are the effective inputs. So, in the first layer of the encoder, only the corresponding connections actually contribute to the encoding process. In decoder, both common genes and scRNA-seq-specific genes are reconstructed and pass through the mask. Then the loss between input and output with both the common genes and specific genes is calculated, and all connections in the last layer contribute to the loss. [In short](javascript:%20void(0)), when training with scRNA-seq data, inClust+ uses common genes to reconstruct common genes and scRNA-seq-specific genes. (**C**) Training inClust+ with MERFISH data. In encoder, only the expression data of common genes are the effective inputs. So, in the first layer of the encoder, only the corresponding connections actually contribute to the encoding process. In decoder, both common gene and scRNA-seq-specific gene are reconstructed, while the scRNA-seq-specific genes are filtered out by the output-mask. Loss is calculated according to the common genes, so only connections corresponding to common genes in the last layer of decoder contribute to the calculation of loss. [In short](javascript:%20void(0)), when training with MERFISH data, inClust+ use common genes to reconstruct common genes. However, after training, inClust+ would output common genes and scRNA-seq-specific genes from the input of common genes.

Figure 4 The diagram for integration of multimodal (triple) datasets by inClust+

(**A**) The workflow of inClust+ for integration of multimodal (triple) datasets (gene expression, protein abundance and chromatin accessibility). Training: ①Generation of the training dataset. To form a training dataset, the data from CITE-seq and ASAP-seq were aligned with shared protein abundance data, and the missing chromatin accessibility data in CITE-seq dataset and missing gene expression data in ASAP-seq dataset were filled with 0. Both multimodal datasets were duplicated 3 times, and concatenated together. ②Generation of the masked-input for the encoder in inClust+. The training dataset multiplies element-wise with an input-mask matrix. The input-mask matrix is as big as the training dataset, and could be divided into six parts. In the first part, the positions of gene expression are filled with 1, and the rest positions are filled with 0. In the second, third, fifth and sixth parts, the position of protein abundance are filled with 1, and the rest positions are filled with 0. In the fourth part, the positions of chromatin accessibility are filled with 1, and the rest positions are filled with 0. The result of multiplication is the masked-input for the encoder, with data from one modality as the input at one time. ③Data encoding, covariates elimination and data integration. The data from different modalities (gene expression, protein abundance or chromatin accessibility) in the masked-input are encoded by the different parts of encoder into the low-dimensional space, and integrated through the constraints of the cell type information. The batch effect is removed by vector subtraction in latent space. ④Reconstruction for data in all three modalities. The decoder simultaneously outputs the reconstructed gene expression, protein abundance and chromatin accessibility. ⑤Generation of the masked-output for loss calculation. The output multiplies element-wise with an output-mask matrix. The output-mask matrix is as large as the output, and could be equally divided into six parts. In the first and the fifth part, the positions of gene expression are filled with 1, and the rest positions are filled with 0. In the second and the third parts, the positions of protein abundance are filled with 1, and the rest positions are filled with 0. In the fourth and sixth parts, the positions of chromatin accessibility are filled with 1, and the rest positions are filled with 0. The result of multiplication is the masked-input for the encoder, with data from one modality as the input at one time. ⑥Calculation of the loss for backpropagation. The MSE between masked-output and the masked-input is calculated as the loss. Data integration: After training, encoded low-dimensional representations are mixed together and clustered according to the cell types without the effect of covariate (batches and modalities). (**B**)-(**D**) Self-reconstruction. (**B**) In the first training phase, due to the influence of mask, only gene expression data is effective for input and output. Therefore, only the corresponding connections in the first layer (upper part) of the encoder and the last layer (upper part) of the decoder actually contribute to the training process. In short, inClust+ uses gene expression data to itself. (**C**) In the second and third training phase, due to the influence of mask, only protein abundance data is effective for input and output. Therefore, only the corresponding connections in the first layer (middle part) of the encoder and the last layer (middle part) of the decoder actually contribute to the training process. In short, inClust+ uses protein abundance data to reconstruct itself. (**D**) In the fourth training phase, due to the influence of mask, only chromatin accessibility data is effective for input and output. Therefore, only the corresponding connections in the first layer (lower part) of the encoder and the last layer (lower part) of the decoder actually contribute to the training process. In short, inClust+ uses chromatin accessibility data to reconstruct itself. (**E**, **F**) alternative-reconstruction. (**E**) In the fifth training phase, due to the influence of mask, only protein abundance data is effective for input and gene expression data is effective for output. Therefore, only the corresponding connections in the first layer (middle part) of the encoder and the last layer (upper part) of the decoder actually contribute to the training process. In short, inClust+ uses protein abundance data to reconstruct gene expression data. (**F**) In the sixth training phase, due to the influence of mask, only protein abundance data is effective for input and chromatin accessibility data is effective for output. Therefore, only the corresponding connections in the first layer (middle part) of the encoder and the last layer (lower part) of the decoder actually contribute to the training process. In short, inClust+ uses protein abundance data to reconstruct chromatin accessibility data.

Figure 6 The diagram for cross-modal generation of inClust+

(**A**) The workflow of inClust+ for integration of two multimodal CITE-seq datasets and a monomodal scRNA-seq dataset, and subsequently cross-modal generation. Training: ①Generation of the training dataset. To form a training dataset, the paired scRNA-seq and CITE-seq data were aligned with shared genes, and the missing protein abundance data in scRNA-seq data were filled with 0. The CITE-seq data were duplicated one time, and concatenated together. ②Generation of the masked-input for the encoder in inClust+. The training dataset multiplies element-wise with an input-mask matrix. The input-mask matrix is as large as the training dataset, and the positions of gene expression data are filled with 1, and the positions of protein abundance data are filled with 0. The result of multiplication is the masked-input of the encoder, and only gene expression data is effective. ③Data encoding, covariates elimination and data integration. Firstly, the gene expression data are encoded in low-dimensional space, and then the batch (covariates) effect is eliminated by subtracting batch information from gene expression data, and the data from different batches are integrated through the constraints of the cell type information and inherent biological characteristic. ④The decoder simultaneously outputs the reconstructed gene expression data and the reconstructed protein abundance data.⑤Generation of the masked-output for loss calculation. The output multiplies element-wise with an output-mask matrix. The output-mask matrix is as large as the output. In the output-mask matrix, the position corresponding to duplicated CITE-seq data could be equally divided into two parts, each part being as big as one CITE-seq data. In the first part, the positions of gene expression data are filled with 1, and the positions of protein abundance data are filled with 0. Alternatively, in the second part, the positions of gene expression data are filled with 0, and the positions of protein abundance data are filled with 1. Meanwhile, the positions of gene expression data in monomodal scRNA-seq data are filled with 1, and the positions of protein abundance data are filled with 0. The result of multiplication is the masked-output. ⑥Calculation of the loss for backpropagation. The MSE between masked outputs and the masked training dataset is calculated as the loss. Label transfer and cross-modal generation: after training, the labels are transferred from cells of multimodal data to the cells of monomodal data in the same clusters. The output of the decoder (step ④) would generate the missing modality in monomodal data. (**B**) Training inClust+ with gene expression data. In these stages, only gene expression data is effective for input and output. Therefore, only the corresponding connections in the first layer (upper part) of the encoder and the last layer (upper part) of the decoder actually contribute to the training process. In short, inClust+ uses gene expression data to reconstruct itself. (**C**) Training inClust+ with gene expression data and translating them into protein abundance data. In these stages, due to the influence of mask, only gene expression data is effective for input and protein abundance data is effective for output. Therefore, only the corresponding connections in the first layer (upper part) of the encoder and the last layer (lower part) of the decoder actually contribute to the training process. In short, inClust+ uses gene expression data to reconstruct protein abundance data.
